# MiR-206 Suppresses Triacylglycerol Accumulation via Fatty Acid Elongase 6 in Dairy Cow Mammary Epithelial Cells

**DOI:** 10.3390/ani14172590

**Published:** 2024-09-06

**Authors:** Xin Zhao, Yu Liu, Yupeng Li, Yuxin Zhang, Chunlei Yang, Dawei Yao

**Affiliations:** Tianjin Key Laboratory of Animal Molecular Breeding and Biotechnology, Tianjin Engineering Research Center of Animal Healthy Farming, Institute of Animal Science and Veterinary, Tianjin Academy of Agricultural Sciences, Tianjin 300381, China; 17852251593@163.com (X.Z.); 17616137227@163.com (Y.L.); 15910772142@163.com (Y.L.); zyx020302@126.com (Y.Z.)

**Keywords:** miR-206, *ELOVL6*, mammary epithelial cells, lipid metabolism

## Abstract

**Simple Summary:**

MiR-206 inhibits lipid production in hepatocytes by promoting insulin signaling pathways. Fatty acid elongase 6 (*ELOVL6*) is involved in fatty acid metabolism in the liver and adipocytes and is critical for lipid synthesis and triacylglycerol accumulation. Data obtained in the present study revealed that miR-206 impacted fatty acid metabolism and the triacylglycerol concentration directly via *ELOVL6* in bovine mammary epithelial cells. Interference with *ELOVL6* inhibited the genes related to fatty acid de novo synthesis and desaturation, fatty acid transport, triacylglycerol synthesis, triacylglycerol hydrolysis, and concentration of long-chain fatty acids. Thus, manipulating the abundance of miR-206 in vivo might be a way to improve the quality of milk.

**Abstract:**

Cow milk possesses high nutritional value due to its rich array of beneficial fatty acids. It is important to understand the mechanisms involved in lipid metabolism in dairy cows. These mechanisms are driven by a complex molecular regulatory network. In addition, there are many regulatory factors involved in the process of fatty acid metabolism, including transcription factors and non-coding RNAs, amongst others. MicroRNAs (miRNAs) can regulate the expression of target genes and modulate various biological processes, including lipid metabolism. Specifically, miR-206 has been reported to impair lipid accumulation in nonruminant hepatocytes. However, the effects and regulatory mechanisms of miR-206 on lipid metabolism in bovine mammary cells remain unclear. In the present study, we investigated the effects of miR-206 on lipid-related genes and TAG accumulation. The direct downstream gene of miR-206 was subsequently determined via a dual-luciferase assay. Finally, the fatty acid content of bovine mammary epithelial cells (BMECs) upon *ELOVL6* inhibition was examined. The results revealed that miR-206 overexpression significantly decreased triacylglycerol (TAG) concentration and abundances of the following: acetyl-coenzyme A carboxylase alpha (*ACACA*); fatty acid synthase (*FASN*); sterol regulatory element binding transcription factor 1 (*SREBF1*); diacylglycerol acyltransferase 1 (*DGAT1*); 1-acylglycerol-3-phosphate O-acyltransferase 6 (*AGPAT6*); lipin 1 (*LPIN1*); and fatty acid elongase 6 (*ELOVL6*). Overexpression of miR-206 was also associated with an increase in patatin-like phospholipase domain-containing 2 (*PNPLA2*), while inhibition of miR-206 promoted milk fat metabolism in vitro. In addition, we found that *ELOVL6* is a direct target gene of miR-206 through mutation of the binding site. Furthermore, *ELOVL6* intervention significantly decreased the TAG levels and elongation indexes of C16:0 and C16:1n-7 in BMECs. Finally, *ELOVL6* siRNA partially alleviated the increased TAG accumulation caused by miR-206 inhibition. In summary, we found that miR-206 inhibits milk fatty acid synthesis and lipid accumulation by targeting *ELOVL6* in BMECs. The results presented in this paper may contribute to the development of strategies for enhancing the quality of cow milk and its beneficial fatty acids, from the perspective of miRNA–mRNA networks.

## 1. Introduction

Cow milk possesses high nutritional value due to its rich types of short- and medium-chain fatty acids, which can help alleviate nutrition malabsorption syndrome, small intestine dysfunction, and hereditary pancreatic disease [1]. In addition, the unsaturated fatty acids in cow milk, such as palmitoleate (16:1 n-7) and oleate (18:1 n-9), which are catalyzed by stearoyl-CoA desaturase 1 (SCD1) and then elongated by fatty acid elongase 6 (ELOVL6), are beneficial for human health [2]. Thus, modification of the levels of beneficial fatty acids in cow milk may be helpful in the implementation of molecular breeding measures for improving milk quality.

MicroRNAs (miRNAs) are endogenous non-coding RNAs (ncRNAs) that participate in the post-transcriptional regulation of gene expression [3]. In general, miRNAs regulate the expression of target genes by binding to the complementary sequence of the 3’ untranslated region (3’-UTR) through its 5’-terminal 2-8 nucleotides to degrade mRNAs or inhibit post-transcriptional gene expression [4]. Previous studies have indicated that many miRNAs play key roles in the regulation of lipid metabolism; these include miR-15b, which negatively correlates with lipid synthesis proteins [5]; miR-126-3p, which inhibits b-casein expression [6]; and miR-200a, which regulates milk fat biosynthesis by targeting IRS2 in bovine mammary epithelial cells (BMECs) [7]. However, the miRNA-mRNA regulatory network involved in the modulation of milk fat metabolism in the mammary glands of ruminants has not yet been well characterized.

miR-206 is known to be one of the tumor suppressor miRNAs whose expression is downregulated in cancer cells. miR-206 can promote apoptosis, induce cell cycle arrest, and inhibit cell migration in various cancers [8]. It was initially considered to be present in muscle cells. In bovine skeletal muscle satellite cells, miR-206 promotes myogenic differentiation and proliferation [9]. A growing body of research has revealed that miR-206 is also involved in the regulation of lipid metabolism. In one study, it was found that the expression level of miR-206 was significantly decreased in bovine mammary gland during the lactation period [10]. miR-206 has also been shown to inhibit de novo lipogenesis, cholesterol synthesis, and gluconeogenesis in mice [11]. Furthermore, glucose-6-phosphate dehydrogenase (G6PD) is a key enzyme of the pentose phosphate pathway, and miR-206 directly binds the 3’-UTR of G6PD mRNA. In another study, it was found that *G6PD* overexpression significantly weakened the inhibition of lipid accumulation and proliferation mediated by miR-206 mimics in hepatocytes [12]. Finally, it is has also been reported that miR-206 directly targets the liver X receptor (*LXRα*) which plays a vital role in the transcriptional regulation of cholesterol metabolism, resulting in a reduction in de novo lipogenesis, VLDL production, and cholesterol synthesis in hepatocytes [13].

Although the above studies indicate that miR-206 plays an important role in the process of lipid metabolism in nonruminants, the specific regulatory mechanism by which miR-206 regulates milk fatty acid metabolism in dairy cows is still unclear. Therefore, the main objective of this study was to analyze the regulatory mechanism governing the effect of miRNAs/mRNAs on fatty acid synthesis. In light of this analysis, we then constructed an miR-206 regulatory signal network. The research reported in this paper provides new ideas which may contribute to the development of future strategies for improving the flavor and quality of milk.

## 2. Materials and Methods

### 2.1. Cell Culture and Transfection

BMECs were donated by Chen Zhi, an associate researcher from the College of Animal Science and Technology, Yangzhou University. Animal collection and protocols were approved by the Animal Care and Use Committee of Institute of Animal Science and Veterinary, Tianjin Academy of Agricultural Sciences. BMECs were cultured in an incubator with a CO_2_ concentration of 5% at 37 °C. The culture medium was composed of DMEM/F12, 10% fetal bovine serum, 5 μg/mL insulin, and 10 kU/mL penicillin/streptomycin. To induce lactogenesis, BMECs used in the experiments described below were cultured in a lactogenic medium for 48 h before the beginning of treatment, in line with previously reported practice [14].

When the cell confluence reached 80%, the siRNA, miRNA-206 mimics, and miRNA-206 inhibitors were transfected into the cells using LipofectamineTM RNAiMAX according to the manufacturer’s instructions, and 3 replicates were created for each group. The medium was changed after 24 h of transfection, and the cells were collected after 48 h of incubation.

### 2.2. RNA Extraction and Real-Time Fluorescence Quantitative PCR

Total RNA was extracted using the TRIzol method and reverse-transcribed into cDNA. Reverse transcription was executed for both miRNA and total RNA. For each sample, 1 μg RNA was used in the reverse transcription, and 50 ng RNA was used in RT-qPCR for each technical duplication. The reactions for miRNA and the total RNA reverse transcription were both carried out according to the TaKaRa kit instructions.

The effects of miR-206 overexpression and *ELOVL6* siRNA intervention on the expression of genes related to lipid metabolism were examined separately via a two-step RT-qPCR. The PCR procedure was as follows: 95 °C predenaturation for 30 s, followed by 95 °C for 5 s, 60 °C for 30 s, and 40 cycles. The PCR mixture was as follows: SYBR 5 μL; 10 µmol/L of upstream and downstream primers, 0.4 μL each; 2 uL of template; and RNase-free water. Finally, the 2^−∆∆Ct^ method was used for relative quantification, with the cycling threshold (Ct) of the double internal reference being the geometric mean of two internal reference genes. Primer sequences were as previously described by Bionaz and Loor [15] (Table 1).

### 2.3. Prediction and Plasmid Construction of miR-206 Target Gene

To explore the mechanism by which miR-206 regulates lipid metabolism, we used the TargetScan 7.1 database (http://www.targetscan.org/vert_71/, accessed on 1 March 2018) to predict the downstream target genes of miR-206. Moreover, the bovine ELOVL6 3’UTR fragment was amplified via PCR and cloned into the XhoI/NotI digestion site of the dual-luciferase reporter gene vector psiCHECK-2 to construct a dual-luciferase reporter gene vector containing the wild-type bovine ELOVL6 3’UTR sequence. Moreover, a dual-luciferase expression vector containing the mutant ELOVL6 3’UTR sequence was constructed by site-directed mutation technique, in which the target of the site-directed mutation was miR-206. The primer sequences are shown in Table 2.

### 2.4. Dual-Luciferase Assays

The cells were co-transfected with miR-206 mimics, inhibitors, and the constructed recombinant vector. Afterward, the cells were completely lysed by adding cell lysis solution and centrifuged at 10,000–15,000× *g* for 3–5 min. The supernatant was then collected for testing. The activities of firefly luciferase and renal luciferase were measured according to the instructions of the dual-luciferase reporter gene detection kit, and the ratio of the two was calculated to represent the relative luciferase activity [14].

### 2.5. ELOVL6 Interference

Three pairs of experimental siRNAs and control siRNAs targeting the CDS of bovine *ELOVL6* were designed using BLOCK-iT™ RNAi Designer (https://rnaidesigner.thermofisher.com/rnaiexpress/, accessed on 1 March 2018) (Table 3) and synthesized by Guangzhou Ribo Biotechnology Co. (Guangzhou, China). The transfection complexes were prepared according to the manufacturer’s instructions, incubated in the dark for 20 min, and then transfected into control and treated mammary epithelial cells at the same time, with three replicates in each group [16]. The expression of the *ELOVL6* was measured via RT-qPCR, and the effect of siRNA intervention of each group was calculated via relative quantification using the 2^−ΔΔct^ method, which was used to isolate the siRNAs with the most pronounced effect.

### 2.6. TAG and Fatty Acid Assays

After the cells were transfected with miR-206 mimics, inhibitors, and *ELOVL6* siRNA, a triacylglycerol assay was performed according to the instructions of the tissue cell triacylglycerol enzyme assay kit. The absorbance at 550 nm was detected using a microplate reader according to the instructions. The TAG standard curve was constructed, and the concentration of TAG in the samples was determined. Finally, the TAG content was corrected for the total protein concentration per mg of cells, in line with previously reported methods [17].

Similarly, after *ELOVL6* siRNA was transfected into the cells for 48 h, the cells were washed three times with PBS; fatty acid extraction and methyl esterification experiments were then carried out, with three replicates in each group. Fatty acid extraction was carried out using the method described by Yao et al. [18].

### 2.7. Statistical Analysis

All data were analyzed using SPSS 19.0 (SPSS Inc., Chicago, IL, USA). The data were expressed as the mean ± SE (standard error) of three independent experiments. The 2^−ΔΔCt^ method was used for relative quantitative analysis. The data were considered statistically significant at *p* < 0.05 (*, *p* < 0.05; **, *p* < 0.01) using Student’s *t*-test (unpaired and two-tailed) or a one-way ANOVA.

## 3. Results

### 3.1. miR-206 Affects Triacylglycerol Levels and Expression of Genes Related to Lipid Metabolism in BMECs

**Figure 1:**
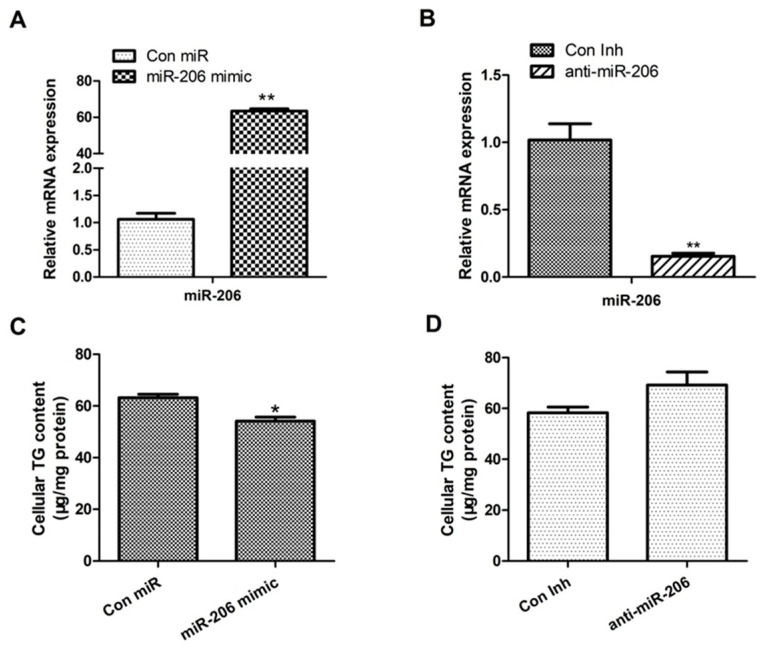
Expression analysis of miR-206 in BMECs. (**A**) Detection of transfection efficiency of miR-206 mimics. (**B**) Detection of transfection efficiency of miR-206 inhibitor. (**C**,**D**) Effect of overexpression or interference of miR-206 on intracellular triglyceride content. **: *p* < 0.01 v. control; * *p* < 0.05 v. control.

To examine the function of miR-206 in BMECs, we designed and synthesized miR-206 mimics and inhibitors and experimentally transfected them. Compared with the control group, the group transfected with the miR-206 mimics presented an approximately 600-fold increase in miR-206 expression (*p* < 0.01; Figure 1A), whereas the group transfected with the miR-206 inhibitors presented an 80% decrease in miR-206 expression (*p* < 0.01; Figure 1B). TAG is the most abundant lipid in milk fat. We measured changes in TAG levels in BMECs and found that the TAG level was significantly reduced in cells overexpressing miR-206 (Figure 1C); however, it did not change significantly when miR-206 expression was inhibited. In conclusion, we found that miR-206 mimics and inhibitors have favorable transfection efficiency, and miR-206 has an important regulatory effect on intracellular lipid accumulation.

**Figure 2:**
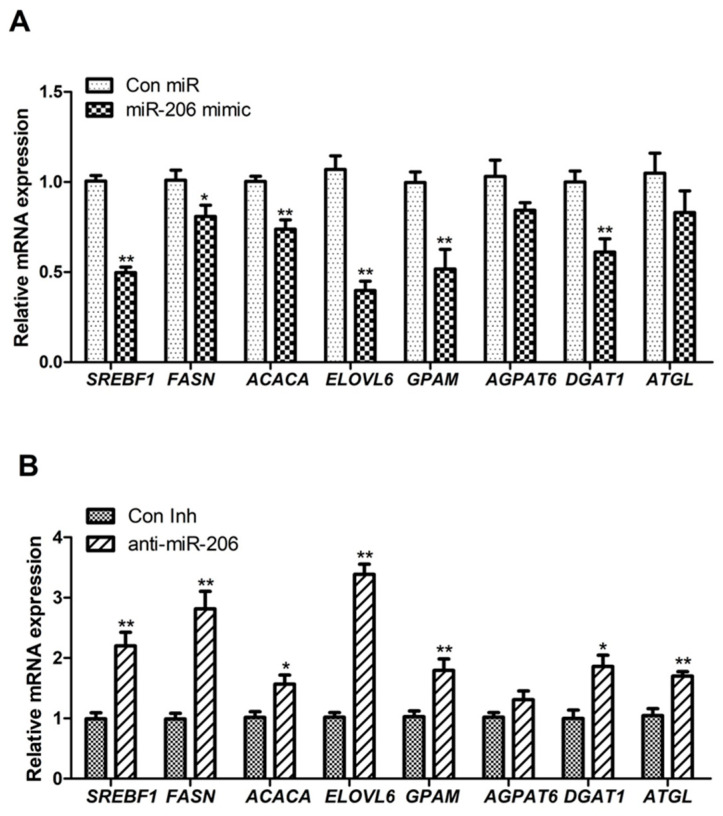
Effect of overexpression or interference of miR-206 on expression of genes related to lipid accumulation and lipid metabolism. (**A**) Variation in genes related to lipid metabolism after overexpression of miR-206. (**B**) Variation in genes related to lipid metabolism after miR-206 inhibition. Quantitative PCR data were calculated using the 2^−ΔΔCt^ method and are presented as mean ± standard error of the means for three independent experiments. ** *p* < 0.01 v. control; * *p* < 0.05 v. control.

Many genes play key regulatory roles in the metabolism of fatty acid, cholesterol, and glucose. In the present study, we used RT-qPCR to examine whether the differential expression of miR-206 could affect the expression of lipid metabolism-related genes. Our results showed that miR-206 overexpression led to highly significant downregulation in the expression of seven lipid metabolism-related genes, namely, *GPAM*, *AGPAT6*, *DGAT1*, *LPIN1*, *SREBF1*, *FASN,* and *ELOVL6* (*p* < 0.01; Figure 2A). In contrast, silencing miR-206 expression significantly upregulated the expression of these genes (*p* < 0.01; Figure 2B).

### 3.2. miR-206 Directly Targets the 3′-UTR of ELOVL6 mRNA

**Figure 3:**
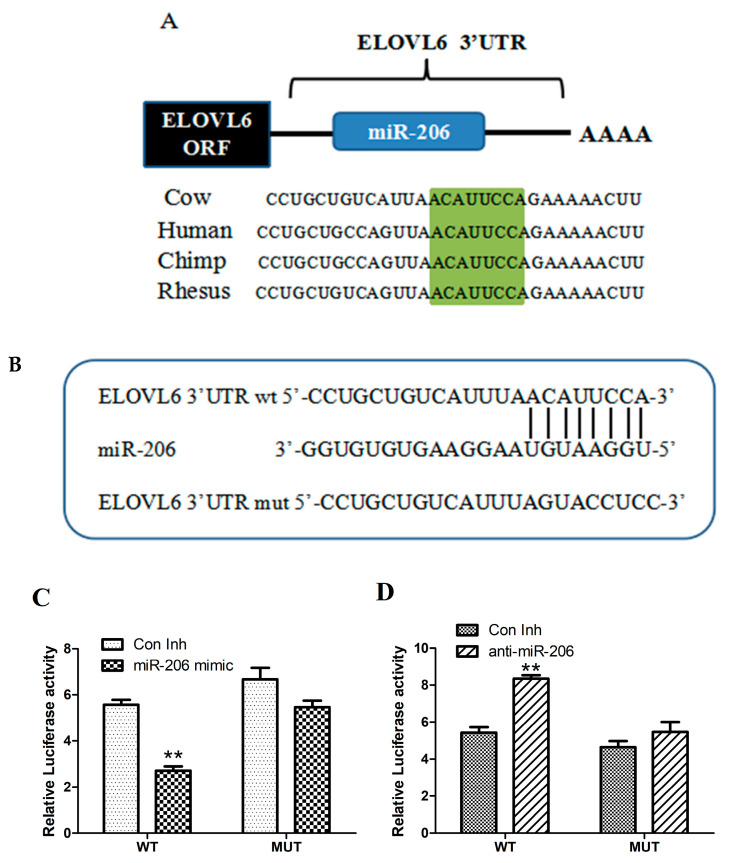
Prediction of miR-206 target genes. (**A**) Analysis of binding sites of miR-206 on *ELOVL6* 3′TR in different species. (**B**) Results of site-directed mutation of miR-206 target sites. (**C**,**D**) Dual-luciferase assay results of miR-206 mimics or inhibitors co-transfected with recombinant vector. ** *p* < 0.01 v. control.

ELOVL6 is an important rate-limiting enzyme in fatty acid synthesis. After overexpressing or silencing miR-206, we found that miR-206 could regulate the expression level of *ELOVL6*. We also discovered that miR-206 is more conserved in humans and chimpanzees (Figure 3A) and that miR-206 binds to the *ELOVL6* 3’UTR (Figure 3B). Consequently, we hypothesized that *ELOVL6* is a potential target gene for miR-206.

To confirm that *ELOVL6* is a direct target of miR-206, we constructed a psiCHECK-2-ELOVL6 3’-UTR recombinant plasmid and carried out luciferase reporter analysis. The results demonstrated that miR-206 overexpression significantly reduced the activity of dual-luciferase reporter gene expression when the *ELOVL6* 3’UTR was a wild type (*p* < 0.05; Figure 3C). Similarly, silencing miR-206 expression significantly increased the activity of dual-luciferase reporter gene expression (*p* < 0.05; Figure 3D). However, miR-206 had no effect on the expression of the dual-luciferase reporter gene when the *ELOVL6* 3’UTR was mutated. Taken together, these findings indicated that miR-206 can act directly on the bovine *ELOVL6* 3’UTR target site.

### 3.3. Interference with ELOVL6 Expression Decreases the Expression of Lipid Metabolism-Related Genes in BMECs

**Figure 4:**
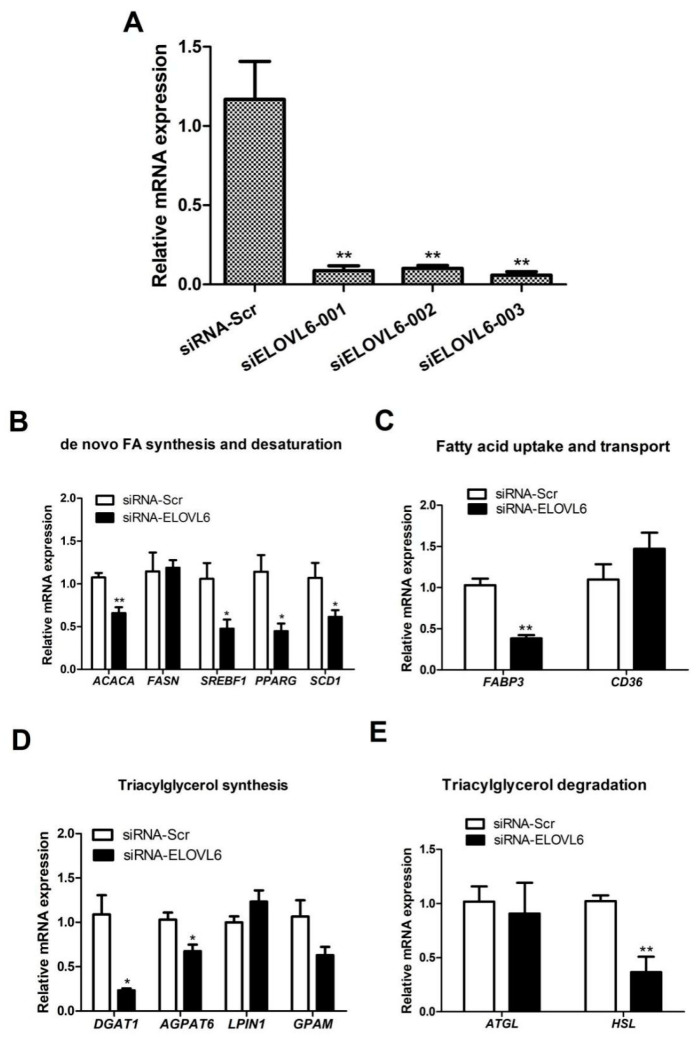
Detection of *ELOVL6* interference efficiency and effect of *ELOVL6* inhibition on genes related to lipid metabolism. (**A**) All three siELOVL6 could significantly interfere with the expression of *ELOVL6*. (**B**) Expression changes in genes related to de novo fatty acid synthesis and desaturation after interference with *ELOVL6*. (**C**) Expression changes in genes related to fatty acid uptake and transport after *ELOVL6* inhibition. (**D**) Expression of TAG synthesis-related genes after *ELOVL6* inhibition. (**E**) Expression of TAG degradation related genes after *ELOVL6* interference. * *p* < 0.05 v. control, ** *p* < 0.01 v. control.

To study the role played by ELOVL6 in BMECs through siRNA, we first selected effective siRNAs. Three pairs of experimental *ELOVL6* siRNA and control siRNA were transfected into mammary epithelial cells, and their transfection efficiency was examined using RT-qPCR. Compared with the control groups, all three *ELOVL6* siRNA RNA groups were found to have highly significant silencing effects (*p* < 0.01; Figure 4A), with *ELOVL6* siRNA-003 having the highest gene-silencing efficiency.

The screened effective *ELOVL6* siRNA was transfected into mammary epithelial cells, and RT-qPCR was used to measure the expression of genes related to milk lipid synthesis. The results suggested that the expression of fatty acid de novo synthesis and desaturation-related genes (*ACACA*, *SREBF1*, *PPARG*, and *SCD1*), fatty acid transport-related genes (*FABP3*), triacylglycerol synthesis-related genes (*DGAT1* and *AGPAT6*), and triacylglycerol hydrolysis-related genes (*HSL*) were significantly decreased (*p* < 0.05; Figure 4). In light of these results, we speculated that ELOVL6 plays an important regulatory role in BMEC lipid metabolism.

### 3.4. Interference with ELOVL6 Expression Affects Intracellular Fatty Acid Composition

**Figure 5:**
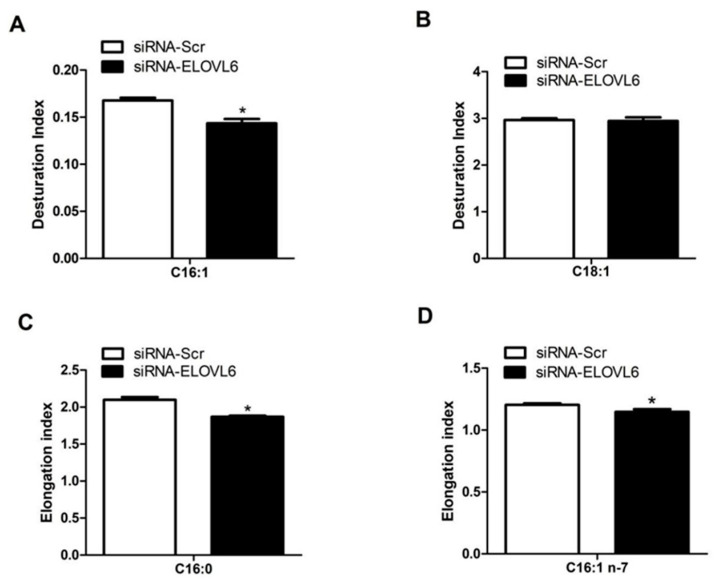
Changes in FA desaturation index and elongation index after overexpression or inhibition of *ELOVL6* in BMEC. (**A**,**B**) Desaturation index of 16:1 and 18:1 after the treatment of siRNA-Scr or siRNA-ELOVL6. (**C**,**D**) Elongation index of 16:0 and 16:1n-7 after treatment with siRNA-Scr or siRNA-ELOVL6. Data are presented as mean ± standard error of the means for 3 individual experiments. * *p* < 0.05 v. control.

**Figure 6:**
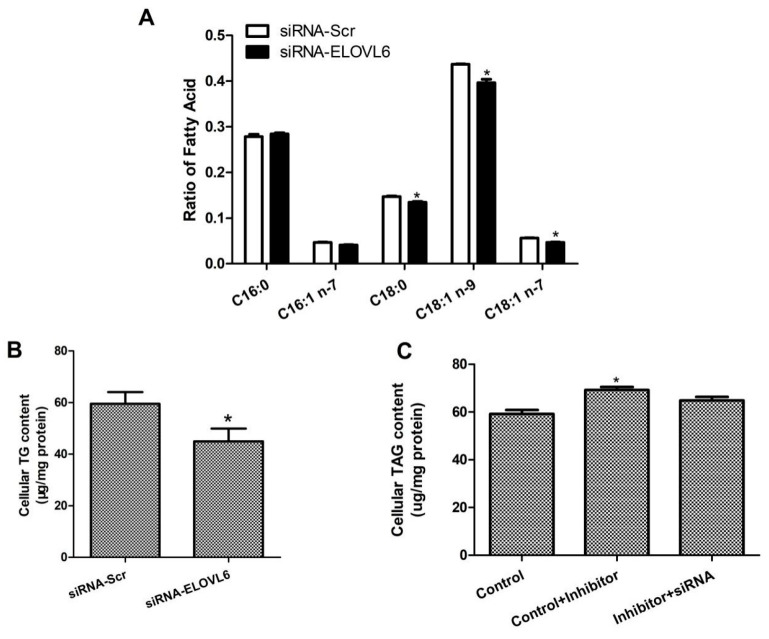
Effects of *ELOVL6* interference on fatty acid composition and triglyceride content in cells. (**A**) Changes in intracellular FA fractions after *ELOVL6* inhibition in BMEC. (**B**) TAG levels in cells transfected with siRNA-Scr or siRNA-ELOVL6. (**C**) TAG levels in cells transfected with negative control inhibitor + control siRNA, inhibitor miR-206+ control siRNA, and inhibitor miR-206+ sirNA-ELOVL6. * *p* < 0.05 v. control.

To further validate the function of ELOVL6, total intracellular fatty acid was extracted and its components were determined using gas chromatography–mass spectrometry. We found that interference with *ELOVL6* expression decreased the desaturation index of palmitoleic acid (C16:1) (*p* < 0.05). In addition, it also significantly decreased the elongation indexes of palmitic acid (C16:0) and palmitoleic acid (C16:1 n-7) (*p* < 0.05; Figure 5). We further investigated the fatty acid fractions and found that the ratios of stearic acid (C18:0), oleic acid (C18:1 n-9), and oleic acid (C18:1 n-7) were significantly lower (*p* < 0.05; Figure 6A). These data suggested that interference with ELOVL6 expression leads to certain dynamic changes in milk fatty acid synthesis in BMECs.

### 3.5. ELOVL6 siRNA Partially Reverses the Decrease in TAG Levels Caused by Inhibition of miR-206

For the above study, we designed three different groups: the miR-206 inhibitor and control siRNA (control); the miR-206 inhibitor and control siRNA (control + inhibitor); and the miR-206 inhibitor and siRNA-ELOVL6 (inhibitor + siRNA). The regulatory role of miR-206 on *ELOVL6* was further demonstrated by detecting changes in the intracellular TAG content. TAG levels were significantly increased when miR-206 was inhibited (control + inhibitor) (*p* < 0.05), but this effect was partially lost with the addition of siRNA-*ELOVL6* (inhibitor + siRNA) (Figure 6C). Consequently, we speculated that inhibition of intracellular miR-206 expression using miR-206 inhibitors increases the expression of endogenous *ELOVL6*, and the addition of siRNA-ELOVL6 counteracts some of the overexpression of *ELOVL6*, resulting in changes in cellular function. In summary, the results of the present study indicated that miR-206 regulates the metabolic process of fatty acids in BMECs through ELOVL6.

## 4. Discussion

miR-206 is a member of the myomiR family of miRNAs (i.e., miR-1, miR-133, and miR-206), which is absent in white adipocytes and specifically expressed in brown adipocytes [19]. MiRNA microarray analysis has shown that miR-206 is significantly downregulated and persistently expressed at low levels throughout adipogenesis under treatment culture conditions [20]. In light of this, we speculated that miR-206 might play an important regulatory role in lipid metabolism. In the present study, we found that miR-206 mimics significantly inhibited lipid accumulation and TAG levels in BMECs. This result is consistent with a reduction in positive Oil Red O staining after miR-206 overexpression in 3T3-L1 cells [21]. In contrast, transfection of hepatoblastoma (HepG2) cells with miR-206 mimics was found to increase lipid accumulation and attenuate triiodothyronine (T3)-induced downregulation of TAG in HepG2 cells [22]. This may have been because the mechanism of action of miR-206 varies in different cells. In conclusion, we demonstrated that miR-206 is involved in the regulation of lipid metabolism and is associated with milk lipid metabolism and secretion.

SREBP1 is central to the transcriptional regulation of mammalian milk fat synthesis [17]. In BMECs, SREBP1 has been shown to play a significant role in the transcriptional regulation of many genes related to milk fat metabolism by overexpression and knockdown [23]. This role was confirmed in the present study. We found that miR-206 overexpression significantly suppressed *SREBF1* expression and knockdown of miR-206 upregulated *SREBF1* expression [24]. A recent survey demonstrated that overexpression of *SREBF1* increased *FASN* expression in GMECs [17]. FASN is the key enzyme in the de novo synthesis of milk fatty acids, as it catalyzes all reaction steps in the synthesis of saturated fatty acids by acetyl-CoA and malonyl-CoA in an NADPH-dependent manner [25]. In the present study, changes in *FASN* expression after promotion or inhibition of miR-206 expression were found to be consistent with changes in SREBP1. In nonruminants, SREBP1 directly controls the expression of *FASN* [26]. In the present study, we again confirmed the fact that *FASN* is a target gene of SREBP1. Previous research has also shown that miR-1/miR-206 inhibits *LXRα* expression at the mRNA and protein levels in hepatocytes, as well as the expression of *ACACA*, a target gene of LXRα [13,26]. In the present study, the downregulation of *ACACA* after overexpression of miR-206 in BMECs was found to be consistent with that observed in hepatocytes. These data suggested that miR-206 may control fatty acid metabolism by regulating the expression of genes related to the de novo synthesis of fatty acids.

In the present study, we also found that the TAG synthesis-related gene *GPAM* was correspondingly down- or upregulated during miR-206 overexpression or disruption, respectively. The GPAM gene promotes the synthesis of TAG in the lipid metabolism pathway [27]. It was indicated that overexpression of miR-223 in intramuscular preadipocytes accelerated cell differentiation and reduced the number of lipid droplets in adipocytes by downregulating *GPAM* expression [28]. Accordingly, controlling the expression of miR-206 may affect the intracellular TAG content as well as the size and number of lipid droplets.

Notably, we found that *ELOVL6* was significantly affected by miR-206 overexpression or silencing. We also discovered that *ELOVL6* is a downstream target gene of miR-206 after software prediction. ELOVL6 is a key regulator of fatty acid composition; it catalyzes the extension of C12-16 saturated and monounsaturated fatty acid chains to form C18 fatty acids, such as stearic acid (C18:0) and oleic acid (C18:1n-9) [29,30]. ELOVL6 has been shown to affect TAG synthesis by altering fatty acid synthesis in buffalo and has been shown to impact the expression of genes related to milk fat metabolism, such as *ACACA* and *FABP3* [2]. The authors of one recent study concluded that knockdown of the ELOVL6 gene significantly increased the expression of the fatty acid desaturase (*SCD1*) and the fatty acid oxidation gene (*CPT1*) and that lipid accumulation in cells was significantly higher [31]. FABP3 is involved in the cellular uptake and metabolism of long-chain fatty acids; it is an intracellular protein which is involved in the transport of fatty acids from the plasma membrane to sites of β-oxidation and triacylglycerol or phospholipid synthesis [32]. In light of these findings, we conjectured that the significant downregulation of FABP3 gene expression might be due to a reduction in the amount of FABP3-bound long-chain fatty acids caused by interference with *ELOVL6* expression.

Previous studies have also revealed that DGAT1 is significantly associated with milk lipid levels [33,34,35]. In one such work, it has shown that overexpression of *DGAT1* in bovine muscle cells significantly increased TAG levels [34]. The final step of TAG synthesis is regulated by DGAT1 and DGAT2 [36]; these catalyze the esterification of acyl-CoA with diacylglycerol (DAG) to form TAG, which then migrates from the endoplasmic reticulum into the cytoplasm to form lipid droplets [37]. In the present study, we observed that downregulation of *ELOVL6* significantly decreased the expression of the DGAT1 gene, and ELOVL6 may indirectly affect TAG synthesis by regulating the expression of TAG synthesis-related genes. In previous studies, it was demonstrated that knockdown of *ELOVL6* decreased the concentrations of C18:1 n-7 and C18:1 n-9 but increased the concentration of C16:0 in GMECs [38]. Conversely, in the present work, we found that downregulation of ELOVL6 gene expression decreased the C16:0 and C16:1 n-7 elongation indices in BMECs by detecting fatty acids, while the C16:1 desaturation index was also significantly downregulated. These findings imply that the ELOVL6 gene is able to regulate fatty acid synthesis in ruminant mammary cells.

Interestingly, C18:1 n-9 is the preferred substrate for the synthesis of TAG [39]. This fatty acid is synthesized in the C18:0 desaturation process. In light of this, we presumed that the function of ELOVL6 is to provide additional substrates for TAG synthesis. Our results revealed that TAG concentrations increased after overexpression of *ELOVL6* and decreased after knockdown of *ELOVL6* in GMECs [38]. This finding was further confirmed in BMECs. A GMEC “rescue” assay then showed that miR-183 inhibited milk lipid synthesis by regulating MST1 [5]. We used the same approach to demonstrate that ELOVL6 siRNA partially reversed the decrease in TAG levels caused by miR-206 overexpression. In summary, we verified that miR-206 regulates milk lipid metabolism in BMECs by targeting *ELOVL6*.

In addition, the fact that miR-206 overexpression is known to impact a broad range of genes involved in lipid metabolism suggests that there may be some potential upstream regulators of miR-206 in BMECs. A study revealed that LncMALAT1 can promote the expression of CD36 through binding with miR-206 in hepatocytes, resulting in increased lipid accumulation [40]. RMRP can bind to miR-206, downregulate its expression and thus promote lipid accumulation by upregulating the PTPN1-PP2ASP1-SREBP1C pathway [41]. Therefore, some upstream regulators or non-coding RNAs may be involved in regulation of miR-206 and subsequently affect the expression of lipid-related genes, ultimately causing changes in lipid metabolism in BMECs.

Although our results prove that miR-206 can regulate milk lipid metabolism by directly targeting *ELOVL6*, there are some limitations in the present study. The mechanisms of action of other genes related to miR-206 regulation of lipid metabolism, such as *FABP3* and *DGAT1*, and the interactions between these genes under miR-206 regulation need to be further explored. Despite these limitations, the findings presented here support the idea that miR-206 plays an important role in mammary lipid metabolism in mammalian ruminants.

## 5. Conclusions

In summary, in the present study, we found that miR-206 plays an important role in lipid metabolism in dairy cow mammary epithelial cells. It can directly bind to *ELOVL6* to regulate the content of long-chain fatty acids in the mammary epithelia cells. However, the upstream regulators and the other downstream genes of miR-206 need to be further explored if a relatively complete signaling pathway in BMECs is to be constructed. Nevertheless, the results presented in this paper may contribute to improving milk quality by regulating the expression of miR-206 or its regulators in the mammary glands of dairy cows.

## Figures and Tables

**Table 1:** Characteristics of the primers used and efficiency of the RT-qPCR reaction.

Gene1	Primer Sequences (5′-3′)	Source
*ACACA*	F: CATCTTGTCCGAAACGTCGAT	[15]
	R: CCCTTCGAACATACACCTCCA	
*AGPAT6*	F: AAGCAAGTTGCCCATCCTCA	[15]
	R: AAACTGTGGCTCCAATTTCGA	
*ATGL*	F: CACCAGCATCCAGTTCAACCT	[15]
	R: CTGTAGCCCTGTTTGCACATCT	
*C* *D36*	F: GTACAGATGCAGCCTCATTTCC	[15]
	R: TGGACCTGCAAATATCAGAGGA	
*DGAT1*	F: CCACTGGGACCTGAGGTGTC	[15]
	R: GCATCACCACACACCAATTCA	
*ELOVL6*	F: TTACAATGGACCTGTCAGCAAAT	[15]
	R: TCATAGTCATAAACCAACCACCC	
*FABP3*	F: GAACTCGACTCCCAGCTTGAA	[15]
	R: AAGCCTACCACAATCATCGAAG	
*FASN*	F: ACCTCGTGAAGGCTGTGACTCA	[15]
	R: TGAGTCGAGGCCAAGGTCTGAA	
*GPAM*	F: GCAGGTTTATCCAGTATGGCATT	[15]
	R: GGACTGATATCTTCCTGATCATCTTG	
*HSL*	F: TCAGTGTCCAAGACAGAGCCAAT	[15]
	R: CATGCAGCTTCAGGCTTTTG	
*LPIN1*	F: TGGCCACCAGAATAAAGCATG	[15]
	R: GCTGACGCTGGACAACAGG	
*MRPL39*	F: AGGTTCTCTTTTGTTGGCATCC	[15]
	R: TTGGTCAGAGCCCCAGAAGT	
*PPARG*	F: CCAAATATCGGTGGGAGTCG	[15]
	R: ACAGCGAAGGGCTCACTCTC	
*SREBF1*	F: CCAGCTGACAGCTCCATTGA	[15]
	R: TGCGCGCCACAAGGA	
*UXT*	F: TGTGGCCCTTGGATATGGTT	[15]
	R: GGTTGTCGCTGAGCTCTGTG	

Annealing temperature for all primers in this table is 60 °C. *UXT*, ubiquitously expressed transcript; *MRPL39*, mitochondrial ribosomal protein L39; *GPAM*, glycerol-3-phosphate acyltransferase; *AGPAT6*, 1-acylglycerol-3-phosphate O-acyltransferase 6; *DGAT1*, diacylglycerol acyltransferase 1; *LPIN1*, lipin 1; *SREBF1*, sterol regulatory element binding factor 1; *FASN*, fatty acid synthase; *ACACA*, acetyl-coenzyme A carboxylase alpha; *ELOVL6*, fatty acid elongase 6; *PPARG*, peroxisome proliferator-activated receptor gamma; *ATGL*, adipose triglyceride lipase; *HSL*, hormone-sensitive lipase; *FABP3*, heart-type fatty acid binding protein 3; *CD36*, thrombospondin receptor.

**Table 2:** Primers used for site-directed mutation of bovine ELOVL6 3′UTR constructs.

Primer Name	Primer Sequences (5′-3′)
*ELOVL6-*3′UTR-F	GATGTTTATCATGGGAGGG
*ELOVL6-*3′UTR-R	GAATTGAAGTGCAGTGGAA
MiR-206 binding site	ACAUUCCA
Mutation site	GUACCUCC
*ELOVL6-mut-F*	CCTGCTGTCATTTAGUACCUCC
*ELOVL6-mut-R*	GCCCAAGTTTTTCGGAGGTAC

**Table 3:** SiRNA sequence of ELOVL6 gene and control group.

Name	Sequence (5′-3′)
siELOVL6-001	GATGTTTATCATGGGAGGG
siELOVL6-002	GAATTGAAGTGCAGTGGAA
siELOVL6-003	ACAUUCCA
siRNA-Scr	GUACCUCC

## Data Availability

The data are available online at https://doi.org/10.6084/m9.figshare.26304157.
